# Dutch norms for the Strengths and Difficulties Questionnaire (SDQ) – parent form for children aged 2–18 years

**DOI:** 10.1186/s12955-018-0948-1

**Published:** 2018-06-14

**Authors:** H. Maurice-Stam, L. Haverman, A. Splinter, H. A. van Oers, S. A. Schepers, M. A. Grootenhuis

**Affiliations:** 1 Psychosocial Department, Paediatrics, Academic Medical Center/Emma Children’s Hospital, G8-142, Postbox 22660, 1100 DD Amsterdam, The Netherlands; 2 Princess Máxima Center for Paediatric Oncology, Utrecht, The Netherlands

**Keywords:** Screening, Psychosocial, Child, Adolescent, Strengths and difficulties questionnaire, Dutch norms

## Abstract

**Background:**

Identification of children at risk for psychosocial problems is important to be able to provide supportive and tailored care at an early stage. Due to its brevity and wide age range, the Strengths and Difficulties Questionnaire (SDQ) Parent Form is an appropriate instrument for use in paediatric clinical practice as it facilitates assessment of psychosocial functioning from young childhood into adulthood. The aim of the present study was to provide Dutch normative data for the SDQ Parent Form.

**Methods:**

A sample of 1947 parents with children aged 2–18 years was drawn from a large panel of a Dutch research agency, stratified on Dutch key demographics of the parents.

The SDQ Parent Form assesses the child’s Emotional symptoms, Conduct problems, Hyperactivity-Inattention, Peer problems and Prosocial behaviour. Summary scores can be calculated: Internalising, Externalising and Total difficulties.

Internal consistency (Cronbach’s alpha coefficient) and normative scores (mean, median, clinical cut-off scores) of the SDQ- Parent Form were calculated in four age-groups 2–3, 4–5, 6–11 and 12–18 years. Gender differences were tested with independent t-tests.

**Results:**

A total of 1174 parents (60.3%) completed the SDQ. In the age-groups 2–3 and 4–5, norm scores are not available for Conduct problems and Peer problems due to insufficient internal consistency. In addition, in age-group 2–3, norm scores for Emotional symptoms and Internalising are not available because of insufficient internal consistency. In the age-groups 6–11 and 12–18, norm scores are available for all scales, with Cronbach’s alpha coefficients 0.53–0.86. The comparison by gender revealed that boys had more behavioural problems than girls (0.000 < *p* < 0.048), most prevalent for Hyperactivity-Inattention, Peer Problems, Prosocial behaviour, Externalising and Total Difficulties.

**Conclusions:**

Dutch normative data by age-group and gender are now available for parent-reported SDQ scores in children aged 2–18 years. Due to insufficient internal consistency, normative scores for 2–5 year-old children could not be presented for several SDQ scales. Yet, the SDQ Total score provides a reliable indication of the psychosocial functioning of younger children. In case of high Total scores in children younger than 6 years, alternatively to scale scores, the answers on the individual items could yield useful clinical information about the child’s problems.

## Background

Thanks to advances in medicine, an increasing number of chronically ill children grow up into adulthood. Therefore, research on the psychosocial consequences of growing up with a chronic disease is of interest. Monitoring and screening in daily clinical care are also important because children with a chronic physical condition were at risk of social-emotional problems [1, 2]. Monitoring and screening enable identification of children at risk for psychosocial problems so that appropriate supportive and tailored care can be provided at an early stage.

An instrument for use in paediatric clinical practice should cover a wide age-range as this facilitates assessment of psychosocial functioning from young childhood into adulthood. Preferably, such an instrument is brief and enables comparison with healthy children and/or children from the general population. The Strengths and Difficulties Questionnaire (SDQ; [3]) is an internationally widely used questionnaire [4–10] that meets these needs. It is a brief behavioural screening questionnaire that covers children’s behaviour, emotions and relationships. As the SDQ focusses on both difficulties and strengths in functioning, it is suitable for use in the general population as well as in populations with a chronic condition. The SDQ has forms for children aged 11–17 years, and for teachers and parents of children aged 2–17 years. All these forms could eventually be used for children up to 19 years if still living with their parents as it has more to do with developmental life stage than with chronological age (communication via email Youth*in*mind, dd 14th November 2014).

The validity of the SDQ is good according to several studies ([11, 12] www.sdqinfo.org), including research in the Netherlands [13–20], though the reliability of some scales appeared to be rather low in preschool-aged children [16]. Several Dutch studies focused on psychometric characteristics of the Dutch SDQ [13–18], but to date, the availability of Dutch norms is limited in terms of age coverage [20] and representativeness [17, 18], and international SDQ research among preschool children is scarce. Community-based norms are required for meaningful interpretation of the scores of chronically ill children because it gives insight in the children’s functioning in comparison with functioning of peers from the general population. In addition, making normative data internationally available can serve research that is focused on populations from different countries and provide more insight into cross-cultural differences. If different countries had different norms, respondents should be compared with their country-specific norm scores, or alternatively, weighted norm scores could be calculated from the country-specific norm scores. The availability of country-specific normative data is especially of interest in psychosocial/QoL research because psychosocial functioning is known to be country- and cultural-specific.

The Parent Form is most suitable for paediatric clinical practice because of its broad age range (2–18 years), from young childhood into adulthood. Therefore, the aim of the present study was to collect normative data from the SDQ Parent Form. Norms are presented in developmental age-groups 2–3 (toddlers), 4–5 (preschoolers), 6–11 (primary schoolers) and 12–18 years (secondary schoolers/teenagers), and separately by gender. In addition, internal consistency of the SDQ is presented in the four age-groups because only measures with sufficient internal consistency are suitable for calculation of normative scores [21].

## Methods

### Procedures and participants

The present study is part of a large study [22, 23] that aimed to collect normative data for several parent-reported questionnaires about psychosocial functioning and health-related quality of life in children and their parents. Data were collected by a Dutch research agency, ‘Taylor Nelson Sofres Netherlands Institute for Public Opinion’ (TNS NIPO), in November and December 2014. For the present study, a sample size of minimal 820 of parents with children aged 2–18 years was planned, based on a sample size of at least 150 parents per age-group with minimal 40 parents represented in each age year. A sample size of 150 per age-group was needed to achieve enough power to detect possible gender differences in each age-group. At least 40 parents per age year was needed to assure that each age year was sufficiently and equally represented in each age-group. In the age-groups 2–3 and 4–5, more than 40 parents per age-year were needed to reach a minimal number of 150 per age-category. In the age-categories 6–11 and 12–18, 40 parents per age-year was decisive for the recruitment. Assuming a response rate of minimal 50%, in total 1640 parents had to be recruited. In the end, TNS NIPO approached 1947 parents to be very sure that the required minimal sample size of 820 would be achieved.

TNS NIPO, a large external research agency, selected parents for the present study to be representative for age, sex, marital status and education of Dutch parents with children in the age range of 2–18 years. TNS NIPO provides access to respondents of TNS NIPObase, a database with a panel of 200,000 respondents who have indicated that they are willing to participate in TNS NIPO research on a regular basis. Respondents were recruited face-to-face or by phone using random sampling methods to ensure representativeness. TNS NIPO uses the software program ‘DIANA’ (www.niposoftware.com) for sampling and weighing procedures. The sample was stratified based on Dutch population figures regarding key demographics (age, sex, marital status and education). A stratified random sampling technique was used to minimize sample variance and increase precision.

One parent per family, able to complete an online Dutch questionnaire, was asked to participate. Prior to the data-collection, TNS NIPO selected the age of the child per family to achieve the desired distribution of the ages. So, the age of the child the parent was asked to complete the SDQ for, was defined by TNS NIPO. The online questionnaire was programmed to prevent missing data. Informed consent was obtained from all participating parents and the study was approved by the Medical Ethics Committee of the Academic Medical Center in Amsterdam. TNS NIPO used the background data of members of the TNS NIPO panel in accordance with the international code of marketing and social research practice of MOA (Center for Marketing Insights Research Analytics) and ESOMAR (European Society for Opinion and Marketing Research).

### Measures

#### Strengths and Difficulties Questionnaire (SDQ)- parent form

The Dutch version of the Strengths and Difficulties Questionnaire - Parent Form for children (SDQ) [3, 18, 24, 25] was used. It consists of 25 items describing positive and negative attributions of children and adolescents that can be allocated to five scales of five items each: Emotional symptoms, Conduct problems, Hyperactivity-Inattention, Peer problems and Prosocial behaviour. Each item has to be scored on a 3-point Likert with 0 ‘not true’, 1 ‘somewhat true’ and 2 ‘certainly true’. Scale scores are computed by summing the scores on the scale items (range 0–10). Higher scores on the Prosocial behaviour scale reflect strengths, whereas higher scores on the other four scales reflect difficulties. A Total difficulty score can be computed by summing the scale scores of the negative attributions; Emotional symptoms, Conduct problems, Hyperactivity-Inattention and Peer problems (range 0–40). In addition, it is possible to calculate scores for Internalising (summing scale scores of Emotional symptoms and Peer problems; range 0–20) and Externalising (summing scale scores of Conduct problems and Hyperactivity-Inattention; range 0–20) [16, 26].

According to Goodman et al. [3, 11] scores above the cut-off (> 90th percentile) are considered ‘abnormal’ (clinical), with the exception of the Prosocial behavior, where scores equal or below the cut-off (≤ 10th percentile) are considered ‘clinical’. Children with clinical scores have an elevated probability of psychiatric disorders. Goodman et al. [26] recommend the use of Internalising and Externalising in low-risk samples, while using the five separate scales when screening for disorders.

There are two versions of the parent form, one for children aged 2–4 years and one for children aged 4–17 years (which can be used for children up to 19 years if still living with their parents; according to the publisher of the SDQ, Youth*in*Mind). The two age versions are identical apart from two items from the scale Conduct problems; for 2–4 years olds ‘lies’ was replaced by ‘argues’, and ‘steals’ was replaced by ‘spite’. The version for children aged 4–17 years was used for the 4 years old children in the current study.

#### Sociodemographic characteristics

Sociodemographic characteristics of children and their parents were provided by TNS NIPO, for participants as well as non-participants: age and gender of children and parents, and parental educational level, country of birth and employment status (see footnotes Table 1).

**Table 1:** Sociodemographic characteristics of participants and non-participants

	Participants		Non-participants		*p*
2–3 years	*N* = 194		*N* = 125		
CHILD					
Age					
M (SD)	3.0 (.6)		3.0 (.6)		.499
range	2.0–3.9		2.0–3.9		
Gender (male) - *% (N)*	51.5	(100)	50.4	(63)	.842
PARENT					
Age					
M (SD)	36.2 (5.3)		35.8 (4.6)		.484
range	26.1–57.6		25.6–52.6		
Gender (male) - *% (N)*	40.7	(79)	45.6	(57)	.390
Country of birth (Netherlands) - *% (N)*	96.4	(186) ^a^	98.4	(123)	.491
Educational level^1^ - *% (N)*					.682
Low	14.4	(28)	13.6	(17)	
Intermediate	44.3	(86)	39.2	(49)	
High	39.7	(77)	46.4	(58)	
Missing	1.5	(3)	.8	(1)	
Paid employment (yes) - *% (N)*	83.0	(161)	90.4	(113)	.071
4–5 years	*N* = 182		*N* = 135		
CHILD					
Age					
M (SD)	5.0 (0.6)		4.9 (0.6)		.331
range	4.0–5.9		4.0–5.9		
Gender (male) - % (*N*)	**61.5**	(112)	**48.1**	(65)	.022
PARENT					
Age					
M (SD)	38.1 (5.0)		37.0 (5.5)		.092
range	27.7–59.1		25.5–57.7		
Gender (male) - *% (N)*	32.4	(59)	42.2	(57)	.078
Country of birth (Netherlands) - *% (N)*	**93.4**	(170)	**97.0**	(131)	.196
Educational level^1^ - *% (N)*					.004
Low	7.7	(14)	17.8	(24)	
Intermediate	40.7	(74)	47.4	(64)	
High	50.5	(92)	34.1	(46)	
Missing	1.1	(2)	.7	(1)	
Paid employment (yes) - *% (N)*	86.3	(157)	81.3	(109) ^a^	.276
6–11 years	N = 403		*N* = 288		
CHILD					
Age					
M (SD)	**8.6** (1.7)		**8.3** (1.8)		.038
range	6.0–11.9		6.0–11.9		
Gender (male) - *% (N)*	50.1	202	54.2	156	.316
PARENT					
Age					
M (SD)	41.0 (5.3)		40.3 (5.2)		.076
range	28.5–61.3		26.5–59.3		
Gender (male) - *% (N)*	39.7	160	45.6	131	.137
Country of birth (Netherlands) - *% (N)*	96.5	389	95.5 ^b^	273	.551
Educational level^1^- *% (N)*					.604
Low	16.4	66	19.8	57	
Intermediate	46.9	189	46.9	135	
High	36.0	145	33.0	95	
Missing	.7	3	.3	1	
Paid employment (yes) - *% (N)*	83.8 ^b^	336	80.1 ^a^	230	.226
12–18 years	*N* = 395		*N* = 225		
CHILD					
Age					
M (SD)	15.5 (2.0)		15.2 (2.0)		.073
range	12.0–18.9		12.0–18.9		
Gender (male) - *% (N)*	54.4	215	52.0	117	.615
PARENT					
Age					
M (SD)	47.3 (5.5)		46.6 (5.7)		.150
range	32.0–75.3		30.6–66.2		
Gender (male) - *% (N)*	39.7	157	41.3	93	.734
Country of birth (Netherlands) - *% (N)*	97.5	385	96.0 ^b^	214	.336
Educational level^1^- *% (N)*					.857
Low	26.8	106	28.0	63	
Intermediate	46.8	185	44.4	100	
High	25.8	102	26.7	60	
Missing	.5	2	.9	2	
Paid employment (yes) - *% (N)*	81.7 ^a^	322	79.6 ^c^	176	.593

^1^Highest level completed: Low: primary education, lower vocational education, lower and middle general secondary education; Intermediate: middle vocational education, higher secondary education, pre-university education; High: higher vocational education, university

^a^unkown for 1 parent

^b^unkown for 2 parents

^c^unkown for 4 parents

Significant differences are presented in bold

### Statistical analysis

To compare sociodemographic characteristics of participants with that of non-participants, independent t-test (age) and Chi-square-test (gender, country of birth, educational level, and employment status) were used.

Cronbach’s alpha coefficients were calculated to assess internal consistency of the SDQ scales, where Cronbach’s alpha coefficients < .50 were considered insufficient, .50–.69 moderate, .70–.79 satisfactory and ≥ .80 good. Cronbach’s alpha coefficients ≥ .70 are recommended for group comparison. For analyzing individual patient scores, Cronbach’s alpha coefficients ≥ .80 are recommended [27]. If scales had insufficient internal consistency (Cronbach’s alpha coefficients < .50), scale scores are not reported for two reasons. First, using scales with low Cronbach’s alpha complicates the interpretation of the scale scores because a low Cronbach’s alpha indicates that scale items do not belong to the same conceptual domain [28]. Second, using scales with low Cronbach’s alpha makes it difficult to detect differences between groups due to large random measurement error [21].

Descriptive statistics were used to calculate normative scores (mean, standard deviation, range, median) in four age-categories (2–3, 4–5, 6–11, 12–18), and seperately by gender. Clinical cut-offs were defined for the five scale scores, the Total difficulty score, and for the scores on Internalising and Externalising (see Measures).

Gender differences regarding the scores on the five scales, the Total difficulty score, and the scores on Internalising and Externalising, were tested with independent t-tests as well as with Mann-Whitney U tests because the distribution of some scale scores was not quite normal. Chi-square tests were used to examine gender differences with regard to the percentage that scored within the clinical range. A significance level of .05 was used for all statistical tests.

The Statistical Package for Social Sciences (SPSS) version 23.0 for Windows was used for all statistical analyses.

## Results

### Participants

Of the 1947 eligible parents, 1174 (60.3%) completed the SDQ. Response rate was 60.8, 57.4, 58.3, 63.7% in the age-groups 2–3, 4–5, 6–11 and 12–18 years respectively. Sociodemographic characteristics of participants and non-participants are presented in Table 1. Participants in the age-groups 2–3 years and 12–18 years did not differ from non-participants regarding their sociodemographic characteristics. In the age-group 4–5 years, the proportion of boys and the educational level of the parents was higher in participants than in non-participants. In the age-group 6–11 years, children of participating parents were older than the children of non-participating parents.

### Internal consistency

Table 2 presents the Cronbach’s alpha coefficients of the SDQ by age-group.

**Table 2:** Internal consistency of the SDQ Parent Form: range, percentage minimum/maximum score, clinical cut-off score

	Cronbach’s α coefficient	Possible range	Min score %	Max score %	Clinical cut-off^d^
2–3 years, *N* = 194					
Emotional symptoms	.45	0–10	–	–	–
5 items					
Conduct problems	.46	0–10	–	–	–
5 items					
Hyperactivity-Inattention	.72	0–10	5.2	.5	> 6
5 item					
Peer problems	.32	0–10	–	–	–
5 items					
Prosocial behaviour	.60	0–10	.0	14.9	< 6
5 items					
Total difficulties^a^	.71	0–40	2.1	.0	> 12
20 items					
Internalizing^b^	.47	0–20	–	–	–
10 items					
Externalizing^c^	.71	0–20	3.6	.0	> 9
10 items					
4–5 years, *N* = 182					
Emotional symptoms	.66	0–10	42.3	.0	> 3
5 items					
Conduct problems	.42	0–10	–	–	–
5 items					
Hyperactivity-Inattention	.80	0–10	13.2	1.1	> 6
5 item					
Peer problems	.48	0–10	–	–	–
5 items					
Prosocial behaviour	.71	0–10	.5	28.0	< 6
5 items					
Total difficulties^a^	.79	0–40	3.3	.0	> 13
20 items					
Internalizing^b^	.67	0–20	26.4	.0	> 5
10 items					
Externalizing^c^	.76	0–20	9.9	.0	> 9
10 items					
6–11 years, *N* = 403					
Emotional symptoms	.76	0–10	27.5	1.0	> 5
5 items					
Conduct problems	.53	0–10	40.2	.0	> 3
5 items					
Hyperactivity-Inattention	.83	0–10	13.2	2.5	> 7
5 item					
Peer problems	.67	0–10	46.7	.0	> 3
5 items					
Prosocial behaviour	.74	0–10	.2	34.2	< 6
5 items					
Total difficulties^a^	.86	0–40	2.5	.0	> 17
20 items					
Internalizing^b^	.80	0–20	17.6	.0	> 8
10 items					
Externalizing^c^	.80	0–20	8.2	.0	> 10
10 items					
12–18 years, *N* = 395					
Emotional symptoms	.70	0–10	33.2	.0	> 4
5 items					
Conduct problems	.62	0–10	48.9	.3	> 2
5 items					
Hyperactivity-Inattention	.82	0–10	18.7	2.3	> 6
5 item					
Peer problems	.68	0–10	38.7	.3	> 4
5 items					
Prosocial behaviour	.71	0–10	.3	22.8	< 6
5 items					
Total difficulties^a^	.83	0–40	3.0	.0	> 14
20 items					
Internalizing^b^	.75	0–20	17.7	.0	> 7
10 items					
Externalizing^c^	.80	0–20	12.4	.0	> 8
10 items					

^a^Total difficulties: emotional problems + conduct problems + hyperactivity/inattention + peer problems

^b^Internalizing: emotional problems + peer problems

^c^Externalizing: conduct problems + hyperactivity/inattention

^d^Scores above the cut-off ( > 90^th^ percentile) are considered ‘clinical’, with the exception of the scale Prosocial where scores equal or below the cut-off (≤ 10^th^ percentile) are considered ‘clinical’

In the age-group *2–3*, the internal consistency of Hyperactivity-Inattention, Total difficulties and Externalising was satisfactory with Cronbach’s alpha coefficients ranging from .71 to .72. The internal consistency of Prosocial behaviour was moderate (Cronbach’s alpha coefficient .60). Internal consistency of the other four scales was insufficient (Cronbach’s alpha coefficients ranging from .32 to 0.47).

In the age-group *4–5*, the internal consistency of Hyperactivity-Inattention, Prosocial behaviour, Total difficulties and Externalising was satisfactory to good (Cronbach’s alpha coefficients .71–.80). Internal consistency of Emotional symptoms and Internalising was moderate (Cronbach’s alpha coefficients .66 and .67). Internal consistency of Conduct problems and Peer problems was insufficient (Cronbach’s alpha coefficients .42 and .48 respectively).

In the age-group *6–11*, the internal consistency was satisfactory to good (Cronbach’s alpas .74–.86), except for Conduct problems and Peer problems, whose internal consistency was moderate (Cronbach’s alpha coefficients .53 and .67 respectively).

In the age-group *12–18*, the internal consistency was satisfactory to good (Cronbach’s alpa coefficients .70–.83), except for Conduct problems and Peer problems, whose internal consistency was moderate (Cronbach’s alpa coefficients .62 and .68 respectively).

### Normative scores

Table 3 presents the normative scores and the percentages in the clinical range in the four age-groups, and seperately by gender. Scores of scales with Cronbach’s alpha coefficients < .50 are not presented.

**Table 3:** Dutch norms for the SDQ Parent form, by age and gender

2–3 years	BOYS *N* = 100				GIRLS *N* = 94				TOTAL *N* = 194				Boys vs girls (*Mean*)		Boys vs girls (% clinical)	
	*Mean*	*SD*	*Median*	*% clinical*	*Mean*	*SD*	*Median*	*% clinical*	*Mean*	*SD*	*Median*	*% clinical* ^a^	*T*	*p*	*Χ* ^*2*^	*p*
Emotional symptoms	–	–	–	–	–	–	–	–	–	–	–	–	–	–	–	–
Conduct problems	–	–	–	–	–	–	–	–	–	–	–	–	–	–	–	–
Hyperactivity-Inattention	3.84	2.18	4.0	11.0	3.62	2.17	3.5	10.6	3.73	2.17	4.0	10.8	.71	.476	.01	.935
Peer problems	–	–	–	–	–	–	–	–	–	–	–	–	–	–	–	–
Prosocial behaviour	***7.34***	1.78	7.0	10.0	***7.87***	1.62	8.0	6.4	7.60	1.72	8.0	8.2	−2.18	.031	.84	.360
Total difficulties^**b**^	8.16	4.07	8.0	12.0	7.43	4.00	7.0	8.5	7.80	4.04	8.0	10.3	1.27	.206	.64	.424
Internalizing^**c**^	–	–	–	–	–	–	–	–	–	–	–	–	–	–	–	–
Externalizing^**d**^	5.83	2.95	6.0	9.0	5.19	3.01	5.0	6.4	5.52	2.99	5.5	7.7	1.49	.137	.47	.495
4–5 years	BOYS *N* = 112				GIRLS *N* = 70				TOTAL *N* = 182				Boys vs girls (*Mean*)		Boys vs girls (% clinical)	
	*Mean*	*SD*	*Median*	*% clinical*	*Mean*	*SD*	*Median*	*% clinical*	*Mean*	*SD*	*Median*	*% clinical* ^a^	*T*	*p*	*Χ* ^*2*^	*P*
Emotional symptoms	1.29	1.68	1.0	9.8	1.19	1.46	1.0	5.7	1.25	1.59	1.0	8.2	.41	.682	.96	.327
Conduct problems	–	–	–	–	–	–	–	–	–	–	–	–	–	–	–	–
Hyperactivity-Inattention	***3.51***	2.47	3.0	13.4	***2.71***	2.53	2.0	11.4	3.20	2.52	3.0	12.6	2.09	.038	.15	.698
Peer problems	–	–	–	–	–	–	–	–	–	–	–	–	–	–	–	–
Prosocial behaviour	7.89	2.02	8.0	***13.4***	8.36	1.59	9.0	***2.9***	8.07	1.88	8.0	9.3	−1.63	.104	5.65	.017
Total difficulties^**b**^	***7.30***	5.00	7.0	11.6	***5.77***	4.38	4.5	5.7	6.71	4.82	5.0	9.3	2.11	.037	1.77	.184
Internalizing^**c**^	2.46	2.53	2.0	9.8	1.87	2.02	1.0	4.3	2.24	2.36	2.0	7.7	1.66	.099	1.86	.173
Externalizing^**d**^	4.84	3.33	4.0	8.0	3.90	3.25	3.0	8.6	4.48	3.32	4.0	8.2	1.87	.063	.02	.898
6–11 years	BOYS *N* = 202				GIRLS *N* = 201				TOTAL *N* = 403				Boys vs girls (*Mean*)		Boys vs girls (% clinical)	
	*Mean*	*SD*	*Median*	*% clinical*	*Mean*	*SD*	*Median*	*% clinical*	*Mean*	*SD*	*Median*	*% clinical* ^a^	*T*	*p*	*Χ* ^*2*^	*p*
Emotional symptoms	2.17	2.30	2.0	***10.4***	1.99	2.10	1.0	***4.5***	2.08	2.18	1.0	7.4	.87	.388	5.12	.024
Conduct problems	1.34	1.52	1.0	7.9	1.12	1.35	1.0	6.5	1.23	1.44	1.0	7.2	1.52	.130	.32	.572
Hyperactivity-Inattention	***3.92***	2.88	3.0	14.9	***3.17***	2.66	3.0	9.0	3.55	2.80	3.0	11.9	2.69	.008	3.34	.068
Peer problems	***1.51***	1.93	1.0	***15.3***	***1.09***	1.52	.0	***8.0***	1.30	1.75	1.0	11.7	2.46	.014	5.34	.021
Prosocial behaviour	8.11	1.93	9.0	***12.4***	8.47	1.79	9.0	***6.5***	8.29	1.87	9.0	9.4	−1.94	.058	4.12	.042
Total difficulties^**b**^	***8.94***	6.70	7.0	***14.9***	***7.37***	5.48	6.0	***5.5***	8.16	6.17	6.0	10.2	2.58	.010	9.70	.002
Internalizing^**c**^	3.69	3.73	2.0	10.4	3.07	3.03	2.0	6.5	3.38	3.41	2.0	8.4	1.81	.071	2.01	.156
Externalizing^**d**^	***5.25***	3.92	4.0	***13.9***	***4.29***	3.42	4.0	***6.0***	4.77	3.71	4.0	9.9	2.62	.009	7.02	.008
12–18 years	BOYS *N* = 215				GIRLS *N* = 180				TOTAL *N* = 395				Boys vs girls (*Mean*)		Boys vs girls (% clinical)	
	*Mean*	*SD*	*Median*	*% clinical*	*Mean*	*SD*	*Median*	*% clinical*	*Mean*	*SD*	*Median*	*% clinical* ^a^	*T*	*p*	*Χ* ^*2*^	*p*
Emotional symptoms	***1.67***	1.87	1.0	9.3	***2.16***	2.10	2.0	13.3	1.89	1.99	1.0	11.1	−2.49	.013	1.61	.205
Conduct problems	1.08	1.57	1.0	14.4	.91	1.17	1.0	11.1	1.00	1.40	1.0	12.9	1.29	.197	.95	.329
Hyperactivity-Inattention	***3.47***	2.67	3.0	***16.3***	***2.38***	2.32	2.0	***7.2***	2.98	2.57	3.0	12.2	4.29	.000	7.53	.006
Peer problems	***1.79***	1.97	1.0	***11.2***	***1.21***	1.62	1.0	***5.6***	1.52	1.84	1.0	8.6	3.24	.001	3.92	.048
Prosocial behaviour	***7.67***	2.04	8.0	***15.8***	***8.36***	1.67	9.0	***6.7***	7.98	1.91	8.0	11.6	−3.73	.000	7.97	.005
Total difficulties^**b**^	***8.01***	5.71	7.0	10.7	***6.66***	5.25	6.0	6.7	7.39	5.54	6.0	8.9	2.44	.015	1.97	.160
Internalizing^**c**^	3.46	3.18	3.0	10.2	3.37	3.19	3.0	11.7	3.42	3.18	3.0	10.9	.28	.782	.21	.649
Externalizing^**d**^	***4.56***	3.64	4.0	***13.0***	***3.29***	2.99	2.0	***6.7***	3.98	3.42	3.0	10.1	3.80	.000	4.35	.037

Scales with too low internal consistency (Cronbach’s alpha < .50) were not analysed; presented with ‘-‘

Significant differences are presented in bold

^a^Scores above the cut-off are considered ‘clinical’, with the exception of the scale Prosocial where scores equal or below the cut-off are considered ‘clinical’ (see Table 2)

Due to the distribution of the scale scores, percentages approach 10.

^b^Total difficulties: emotional problems + conduct problems + hyperactivity/inattention + peer problems

^c^Internalizing: emotional problems + peer problems

^d^Externalizing: conduct problems + hyperactivity/inattention

In the age-group *2–3*, boys scored lower than girls (*p* < .05) on Prosocial behaviour.

In the age-group *4–5*, boys scored higher than girls on Hyperactivitiy-Inattention and Total difficulties (*p* < .05). More boys than girls scored in the clinical range of Prosocial behaviour (13.4% vs 2.9%, *p* < .05).

In the age-group *6–11*, boys scored higher than girls on Hyperactivity-Inattention (*p* < .01), Peer problems (*p* < .05), Total difficulties (*p* < .05) and Externalising (*p* < .01). Significantly more boys than girls scored in the clinical range of Emotional symptoms (10.4% vs 4.5%, *p* < .05), Peer problems (15.3% vs 8.0%, *p* < .05), Prosocial behaviour (12.4% vs 6.5%, p < .05), Total difficulties (14.9% vs 5.5%, *p* < .01) and Externalising (13.9% vs 6.0%, *p* < .01).

In the age-group *12–18*, boys scored lower than girls on Emotional symptoms (*p* < .05) and Prosocial behaviour (*p* < .001), and higher on Hyperactivity-Inattention (*p* < .001), Peer problems (*p* < .01), Total difficulties (*p* < .05) and Externalising (p < .001). In addition, significantly more boys than girls scored in the clinical range of Hyperactivity-Inattention (16.3% vs 7.2%, p < .01), Peer problems (11.2% vs 5.6%, *p* < .05), Prosocial behaviour (15.8% vs 6.7%, p < .01) and Externalising (13.0% vs 6.7%, *p* < .05).

The results of the independent t-tests were confirmed by the Mann-Whitney U–tests.

## Discussion

This study provides Dutch normative parent-reported SDQ scores for children aged 2–18 years, disaggregated by age-groups 2–3, 4–5, 6–11 and 12–18 years. Dutch norms are largely in line with norms from other countries in Europe [4–9] (www.sdqinfo.org). Finland is an exception to this because Finnish norm scores are lower [10]. Cultural differences and a high level of social welfare and well-being of children in Finland were given as possible explanations [9]. However, a really proper comparison was not possible because the normative samples differed in age categories. Moreover, norms for children younger than 4 years of age are scarce (http://www.sdqinfo.org).

In the age-groups 6–11 and 12–18, internal consistency of most scales is sufficient for group comparisons, while Hyperactivity-Inattention, Externalising, Internalising (only in the 6–11 age-group) and Total Difficulties had internal consistency (Cronbach’s alpha coefficients ≥ .80) good enough for use at the individual level. The internal consistencies were in line with Cronbach’s alpha coefficients reported by Goodman [11], with those reported in previous Dutch studies [14, 17, 18] and with those reported in a review of 48 studies about the psychometric characteristics of the SDQ used over the world [12]. In this review, the weighted mean internal consistency of the (sub)scales of the SDQ parent form used in children aged 4–12 years ranged from 0.53 (Peer problems) to 0.80 (Total Difficulties).

Unfortunately, in the age-groups 2–3 and 4–5, not all scores could be presented because of insufficient internal consistency: Conduct problems and Peer problems in both age-groups, and Emotional problems and Internalising in the youngest age-group. The rather low internal consistency of the SDQ we found in young children is in accordance with findings from previous research among preschool children who visited a Center for Preventive Child Healthcare in the Netherlands [15, 16, 19] and among children of age 4–7 years in the Netherlands [20]. To date, other studies about psychometric characteristics of the SDQ used in children younger than 4 years were not published. Perhaps the low internal consistency of some SDQ scales is due to the fact that several items do not apply well to the developmental phase young children are in (e.g. “considerate of other people’s feelings”) or could not be considered as problematic behaviour in toddlers (e.g. “Often has temper tantrums or hot tempers”). Therefore, caution is warranted for the use of the SDQ in young children, especially for the use of screening for disorders. Even though high Total difficulty scores indicate psychosocial problems, the low internal consistency of several scales does not justify decisions about further psychological treatment [15]. In case of high Total scores in children younger than 6 years, we recommend to further examine the answers on the individual items because these could yield useful clinical information about the child’s problems, alternatively to the scale scores. This is a common procedure in clinical practice to get more detailed insight in the child’s problems. Nevertheless, one should wonder whether it is reasonable to assume that behavioral-emotional functioning could be assessed appropriately in the whole pediatric age-range with the same instrument. More research on the validity of the SDQ among young children is needed as validity studies in the youngest age group are scarce.

The comparison by gender revealed that, especially in the age-groups 6–11 and 12–18, boys had less favourable scores than girls. The gender differences were most prevalent for Hyperactivity-Inattention, Peer Problems, Prosocial behaviour, Externalising and Total Difficulties. These gender differences were in line with results from previous research among Dutch children aged 8–16 years [17, 18] and were also in line with normative data from other European countries [5–8]. Furthermore, we found that girls aged 12–18 had more Emotional symptoms than boys. Previous studies on parent-reported emotional problems in children yielded mixed results [2, 18] [5–8], while to date, in the age category 12–18 years no SDQ norming studies were published. Because of the gender differences, it is recommended to use gender-matched normative scores.

Some limitations should be noticed. It is questionable whether the sample size was large enough for gender-specific normative data. Especially in the youngest age-groups, the standard deviation of some normative values was large relative to the mean scale score. Furthermore, sample size was not large enough to define clinical cut-off scores that corresponded exactly with the 10th or 90th percentile.

The second limitation concerned the representativeness of the sample. Although the current data is limited to participants with internet access, the fact that 97% of households in the Netherlands have access to the internet [29], suggests that the online version of the SDQ can be safely used for the general Dutch population. The response rate of 60% was not optimal. Though few sociodemographic differences were found between respondents and non-respondents, highly educated parents and parents born in the Netherlands might be slightly over-represented, as is the case in most studies. Explorative analyses (data not shown) indicated that low parental education level was associated with more problems in their children as measured with the SDQ Parent Form. Therefore, the results of the current study might present an underestimation of psychosocial problems among children in the Netherlands. Further research is needed to explain the relationship between parental educational level and psychosocial problems in children. A third limitation is that it was not possible to calculate normative scores by health condition (healthy, physical, mental) because it was with the current data not possible to distinguish between physical and mental health conditions in a reliable way.

It is a strength of the study that besides mothers, a considerable part of the respondents consisted of fathers. It would be interesting to examine whether the fathers’ perspective of the child’s behaviour differs from the perspective of the mothers. Unfortunately, the present study data was not appropriate to examine differences between father- and mother report because mothers and fathers were from different families. To address this interesting issue, it is recommended to include fathers and mothers from the same child in future studies.

## Conclusions

Dutch normative data by age-group and gender are now available for parent-reported SDQ scores in children aged 2–18 years. Due to insufficient internal consistency, normative scores for 2–5 year-old children could not be presented for several SDQ scales. Yet, the SDQ Total score provides a reliable indication of the psychosocial functioning of younger children. In case of high Total scores in children younger than 6 years, alternatively to the scale scores, the answers on the individual items could yield useful clinical information about the child’s problems.

## References

[1] Pinquart M, Shen Y (2011). Behavior problems in children and adolescents with chronic physical illness: a meta-analysis. J Pediatr Psychol.

[2] Glazebrook C, Hollis C, Heussler H, Goodman R, Coates L (2003). Detecting emotional and behavioural problems in paediatric clinics. Child Care Health Dev.

[3] Goodman R (1997). The strengths and difficulties questionnaire: a research note. J Child Psychol Psychiatry.

[4] Goodman R (1999). The extended version of the strengths and difficulties questionnaire as a guide to child psychiatric caseness and consequent burden. J Child Psychol Psychiatry.

[5] Niclasen J, Teasdale TW, Andersen AM, Skovgaard AM, Elberling H, Obel C (2012). Psychometric properties of the Danish strength and difficulties questionnaire: the SDQ assessed for more than 70,000 raters in four different cohorts. PLoS One.

[6] Smedje H, Broman JE, Hetta J, von Knorring AL (1999). Psychometric properties of a Swedish version of the “strengths and difficulties questionnaire”. Eur Child Adolesc Psychiatry.

[7] Tobia V, Marzocchi GM. The strengths and difficulties questionnaire-parents for Italian school-aged children: psychometric properties and norms. Child Psychiatry Hum Dev. 2018;49:1–8. 10.1007/s10578-017-0723-2 28391540

[8] Woerner W, Becker A, Rothenberger A (2004). Normative data and scale properties of the German parent SDQ. Eur Child Adolesc Psychiatry.

[9] Borg AM, Kaukonen P, Joukamaa M, Tamminen T (2014). Finnish norms for young children on the strengths and difficulties questionnaire. Nord J Psychiatry.

[10] Koskelainen M, Sourander A, Kaljonen A (2000). The strengths and difficulties questionnaire among Finnish school-aged children and adolescents. Eur Child Adolesc Psychiatry.

[11] Goodman R (2001). Psychometric properties of the strengths and difficulties questionnaire. J Am Acad Child Adolesc Psychiatry.

[12] Stone LL, Otten R, Engels RC, Vermulst AA, Janssens JM (2010). Psychometric properties of the parent and teacher versions of the strengths and difficulties questionnaire for 4- to 12-year-olds: a review. Clin Child Fam Psychol Rev.

[13] Crone MR, Vogels AG, Hoekstra F, Treffers PD, Reijneveld SA (2008). A comparison of four scoring methods based on the parent-rated strengths and difficulties questionnaire as used in the Dutch preventive child health care system. BMC Public Health.

[14] Vogels AG, Crone MR, Hoekstra F, Reijneveld SA (2009). Comparing three short questionnaires to detect psychosocial dysfunction among primary school children: a randomized method. BMC Public Health.

[15] Theunissen MH, Vogels AG, de Wolff MS, Crone MR, Reijneveld SA (2015). Comparing three short questionnaires to detect psychosocial problems among 3 to 4-year olds. BMC Pediatr.

[16] Theunissen MH, Vogels AG, de Wolff MS, Reijneveld SA (2013). Characteristics of the strengths and difficulties questionnaire in preschool children. Pediatrics.

[17] Muris P, Meesters C, van den Berg F (2003). The strengths and difficulties questionnaire (SDQ)--further evidence for its reliability and validity in a community sample of Dutch children and adolescents. Eur Child Adolesc Psychiatry.

[18] van Widenfelt BM, Goedhart AW, Treffers PD, Goodman R (2003). Dutch version of the strengths and difficulties questionnaire (SDQ). Eur Child Adolesc Psychiatry.

[19] Mieloo CL, Bevaart F, Donker MC, van Oort FV, Raat H, Jansen W (2014). Validation of the SDQ in a multi-ethnic population of young children. Eur J Pub Health.

[20] Stone LL, Janssens JM, Vermulst AA, Van Der Maten M, Engels RC, Otten R (2015). The strengths and difficulties questionnaire: psychometric properties of the parent and teacher version in children aged 4-7. BMC Psychol.

[21] Ponterotto JG, Ruckdeschel JC (2007). An overview of coefficient alpha and a reliability matirx for estimating adequacy of internal consistency coefficients with psychological research measures. Percept Mot Skills.

[22] van Oers HA, Schepers SA, Grootenhuis MA, Haverman L. Dutch normative data and psychometric properties for the distress thermometer for parents. Qual Life Res. 2017;26(1):177–82. 10.1007/s11136-016-1405-4 PMC5243897 27589979

[23] Schepers SA, van Oers HA, Maurice-Stam H, Huisman J, Verhaak CM, Grootenhuis MA, Haverman L (2017). Health related quality of life in Dutch infants, toddlers, and young children. Health Qual Life Outcomes.

[24] Goedhart A, Treffers F, van Widenfelt B (2003). Vragen naar psychische problemen bij kinderen en adolescenten: De Strenghts and difficulties questionnaire (SDQ). [questions about mental problems in children and adolescents: the Strengtha and difficulties questionnaire]. Maanblad Geestelijke Volksgezondheid.

[25] Goodman R, Meltzer H, Bailey V (1998). The strengths and difficulties questionnaire: a pilot study on the validity of the self-report version. Eur Child Adolesc Psychiatry.

[26] Goodman A, Lamping DL, Ploubidis GB (2010). When to use broader internalising and externalising subscales instead of the hypothesised five subscales on the strengths and difficulties questionnaire (SDQ): data from British parents, teachers and children. J Abnorm Child Psychol.

[27] Nunnally JC, B IR. Psychometric Theory. 3rd ed. New York: McGraw-Hill; 1994.

[28] Bowling A. Research methods in health: Investigating health and health services. Berkshire: Open University Press; 2007. p147–56.

[29] Statistieken over de digitale economie en de samenleving - huishoudens en personen [Statistics about digital economy and society**].** [http://ec.europa.eu/eurostat/statistics-explained/index.php/Digital_economy_and_society_statistics_-_households_and_individuals/nl#Internettoegang].

